# Subtypes of Sport-Related Concussion: a Systematic Review and Meta-cluster Analysis

**DOI:** 10.1007/s40279-020-01321-9

**Published:** 2020-07-27

**Authors:** S. Langdon, M. Königs, E. A. M. C. Adang, E. Goedhart, J. Oosterlaan

**Affiliations:** 1 Emma Children’s Hospital, Amsterdam UMC (Location Academic Medical Center), University of Amsterdam, Emma Neuroscience Group, Meibergdreef 9, 1105 AZ Amsterdam, The Netherlands; 2 Sports Medical Centre, Royal Netherlands Football Association (KNVB), Woudenbergseweg 56-58, 3707 HX Zeist, The Netherlands

## Abstract

**Objective:**

Current clinical guidelines provide a unitary approach to manage sport-related concussion (SRC), while heterogeneity in the presentation of symptoms suggests that subtypes of SRC may exist. We systematically reviewed the available evidence on SRC subtypes and associated clinical outcomes.

**Data Sources:**

Ovid Medline, Embase, PsycINFO, and SPORTDiscus

**Eligibility Criteria for Selecting Studies:**

Electronic databases were searched for studies: (i) identifying SRC symptom clusters using classification methodology; or (ii) associating symptom clusters to clinical outcome variables. A total of 6,146 unique studies were identified, of which 75 full texts were independently assessed by two authors for eligibility. A total of 22 articles were included for systematic review.

**Data Extraction:**

Two independent authors performed data extraction and risk of bias analysis using the Cochrane Collaboration tool.

**Data Synthesis:**

Six studies found evidence for existence of SRC symptom clusters. Combining the available literature through Multiple Correspondence Analysis (MCA) provided evidence for the existence of a *migraine* cluster, a *cognitive–emotional* cluster, a *sleep–emotional* cluster, a *neurological* cluster, and an *undefined feelings* cluster. Nineteen studies found meaningful associations between SRC symptom clusters and clinical outcomes. Clusters mapping to the *migraine* cluster were most frequently reported in the literature and were most strongly related to aspects of clinical outcome.

**Conclusions:**

The available literature provides evidence for the existence of at least five subtypes in SRC symptomatology, with clear relevance to clinical outcome. Systematically embedding the differentiation of SRC subtypes into prognosis, clinical management, and intervention strategies may optimize the recovery from SRC.

**Electronic supplementary material:**

The online version of this article (10.1007/s40279-020-01321-9) contains supplementary material, which is available to authorized users.

## Key Points

**Table:** 

This systematic review and meta-cluster analysis provides robust evidence for the existence of at least five SRC subtypes, identified as a migraine cluster, a *cognitive–emotional* cluster, a *sleep–emotional* cluster, a *neurological* cluster, and an *undefined feelings* cluster, with clear relevance to clinical outcome.
The results of this study may pave the way for the transition from a unitary approach to SRC management towards individualized and targeted concussion management and treatment, with the ultimate aim to optimize the recovery of SRC.

## Introduction

In the United States alone, an estimated 2.87 million individuals seek care at the Emergency Department for traumatic brain injury each year [1], among which ~ 90% are individuals presenting with mild traumatic brain injury (TBI, i.e. concussion; 2). Concussion as sustained during sports participation accounts for a considerable number of TBI cases, up to 45% in children [3]. A sport-related concussion (SRC) is potent to produce a constellation of acute and post-acute symptoms, incapacitating athletes to return to sport practices [4]. SRC is a heterogeneous injury in terms of etiology and pathophysiology [5, 6] and consequently involves highly variable presentations of symptoms [7]. While current clinical guidelines provide a unitary approach to manage SRC, the heterogeneity in the presentation of SRC symptoms suggests that subtypes of SRC may exist. Subtypes of SRC may be associated with differential clinical outcomes that may require subtype-specific clinical management and treatment.

Diversity in etiology and pathophysiology is thought to be one source of heterogeneity in symptom presentation. SRC may be caused by a direct blow to the head, face, neck, or elsewhere to the body, with linear and rotational acceleration–deceleration forces acting on the brain [8]. Disturbances have been observed at several levels of brain structure and function, among which cellular functioning [9], white matter integrity [10], and functional connectivity [11]. These pathophysiological changes occur diffusely in the brain, while variations in injury characteristics (e.g. force, impact location and direction of skull acceleration–deceleration and rotation) may contribute to marked heterogeneity in SRC phenotypes [4, 5]. The heterogeneity in SRC is also reflected in at least three aspects of clinical outcome: (i) the type of SRC symptoms and functional impairments, (ii) the evolvement of symptoms and impairment over time, and (iii) the recovery trajectory duration.

Athletes that sustained a SRC may experience a wide range of subjectively reported symptoms [4], such as physical symptoms (e.g. dizziness, headache), cognitive symptoms (e.g. difficulty concentrating and feeling mentally foggy), sleep/wake-related symptoms (e.g. drowsiness, insomnia), and affective symptoms (e.g. sadness, anxiety). Likewise, a range of objectively measured impairments have been identified after SRC, such as vestibular impairments (e.g. gait unsteadiness), oculomotor impairments (e.g. blurred vision), physical impairments (e.g. amnesia, loss of consciousness), and cognitive impairments (e.g. slowed information processing). The range of potential symptoms and impairments after SRC give rise to a highly individualized nature of SRC-related sequelae [4].

Over time, the presentation of SRC symptoms may also vary during the course of recovery [4, 12]. Certain consequences typically present as on-field impairments immediately after the sustained injury (e.g. loss of consciousness or post-traumatic amnesia), while other symptoms may not become apparent in the first several hours or even days post-injury. Likewise, the duration of recovery from SRC is also subject to distinct variability between athletes [4]. On average athletes recover spontaneously around 10–14 days after concussion [13] with 80–90% experiencing full recovery within one month [13–15]. Nevertheless, around 10% of athletes with a SRC remain symptomatic for more than 3 months [16] and are typically diagnosed with persisting symptoms after concussion [17]. It has been shown that the presence of specific symptoms and/or impairments is related to the length of the recovery timeframe and the risk of persisting symptoms after concussion [18–20]. Taken together, these findings indicate that in addition to variability in the type and severity of SRC consequences between athletes, there is also notable inter-individual variability in the emergence, evolution, and recovery of symptoms and impairments over time [21].

The evidence on the heterogeneity of etiology and pathophysiology in SRC, resulting in differential symptom presentations and recovery trajectories, indicates that SRC may involve distinct subtypes in which specific symptoms and impairments cluster together. In line with this idea, Collins and colleagues proposed a practice-based model on the delineation of SRC subtypes based on the characteristic symptoms observed at 1 week post-injury, differentiating six subtypes characterized by cognitive/fatigue symptoms, vestibular symptoms, ocular-motor symptoms, anxiety/mood symptoms, post-traumatic migraine symptoms. and cervical symptoms, respectively [22]. Although compelling, the model by Collins and colleagues is practice-based and, therefore, sensitive for bias in the conceptualization of subtypes. Therefore, the model awaits testing in an overview of empirical evidence focusing on data-driven clustering of symptoms into SRC subtypes.

This systematic review aims to evaluate and integrate all available evidence on the classification of SRC symptoms into clusters. Considering that the presentation of particular symptoms is related to prolonged recovery, it is likely that potential subtypes of SRC also relate to differential recovery trajectories. Therefore, we also aim to evaluate the literature on the relation between symptom clusters and clinical outcome. Thereby, this study will reveal the state of the literature with regard to the existence of SRC symptom subtypes and their clinical relevance. The results of this study may pave the way for the transition from a unitary approach to SRC management towards individualized and targeted concussion management and treatment, with the ultimate aim to optimize the recovery of SRC and prevent the development of persisting symptoms after concussion.

## Methods

This study was performed according to the guidelines set forth by the Meta-analysis Of Observational Studies in Epidemiology (MOOSE) group for reporting of systematic reviews of observational studies [23].

### Study Search and Selection

#### Eligibility Criteria

Studies were considered eligible for this systematic review if they reported on athletes with SRC, and (i) identified SRC symptom clusters using classification methodology, or (ii) examined the association of SRC symptom clusters with clinical outcome variables (e.g. recovery timeframe). Articles were excluded if they: (i) were not peer-reviewed or (ii) represented abstracts of congress presentations.

#### Information Sources

The search strategy was designed in collaboration with the Amsterdam University Medical Centers (Academic Medical Center location) librarian and involved the following combinations of search terms and their equivalents: *Concussion* AND *Sport* AND *Symptom assessment* (see Online Resource 1 for the specific search queries). The search was performed in the electronic databases Ovid MEDLINE, Embase, PsycINFO, and SPORTDiscus, using both simple search terms and hierarchical family forms (e.g., Medical Subject Headings) and covered all entries between 1946 and 19 December 2018. Furthermore, the reference lists of included articles were hand-searched for additional articles satisfying the inclusion criteria.

### Study Selection

The retrieved records were deduplicated and subsequently screened for eligibility by two reviewers (E.A. and S.L.) based on title and abstract. Relevant records were then independently assessed by the two reviewers based on full texts. Differences in study selection between reviewers were solved by consensus between authors E.A. and S.L.

### Data Extraction

Included studies were systematically reviewed and the following information was extracted from the articles: (i) study design, (ii) study samples, (iii) sample size of patients with SRC and controls, (iv) time of symptom assessment, (v) assessment tools, (vi) methods of analysis, (vii) identified symptom clusters and associations between symptom clusters, and (ix) clinical outcome variables as reported by the authors. Studies identifying SRC symptom clusters using classification methodology were divided in subsections according to their methods of analysis: (1) *explorative*, data-driven identification of clusters, (2) *explorative and confirmative*, data-driven identification of clusters and subsequently verification of these clusters, and (3) *supportive*, collapsing symptoms into clusters as determined by prior research or based on theory/hypothesis and subsequent verification of these clusters in current samples. To provide a systematic aggregation of the available literature, a Multiple Correspondence Analysis (MCA) for clustering of binary data was then conducted on the reported results of SRC symptom clustering using the ‘FactoMineR’ package in R [24, 25]. More specifically, we inserted SRC symptoms as cases in the MCA, while the identified clusters were used as grouping variables. By this procedure, MCA identified clusters of symptoms corresponding to the same grouping variables across the literature. The number of clusters to extract was determined in the eigenvalues histogram. Subsequently, each extracted cluster was labeled according to the set of symptoms that made the strongest contribution to the cluster in terms of effect size (*ƞ*^2^). The boundary of this set of symptoms was set at the largest drop in *ƞ*^2^ between two subsequent variables in the scree plot (also see Online Resource 2). Clusters from the literature that were found to be associated with clinical outcomes were matched to MCA-identified clusters, based on the highest match in overlapping symptoms between clusters (if the studied cluster contained ≥ 50% of symptoms in one of the MCA clusters).

If available, standardized effect sizes (Cohen’s d, Pearson *r* correlation) were calculated for the reported effects and interpreted according to Cohen’s guidelines for small (*d* = 0.2–0.5, *r* ± 0.1), medium (*d* = 0.5–0.8, *r* ± 0.3), and large (*d* ≥ 0.80, *r* ≥ 0.5) effect sizes.

### Risk of Bias Analysis

Two independent authors (E.A. and S.L.) assessed the quality of the included studies using the Cochrane Collaboration Tool [26]. As suggested in the handbook, this tool was adapted to enable risk of bias assessment in observational studies. Risk of bias was assessed in terms of selection bias (i.e. representative patient group and adequate case definition), detection bias (i.e. outcome assessor blinding), performance bias (i.e. outcome patient blinding and outcome objectivity), follow-up bias (i.e. follow-up measurements and attrition), and other bias (analysis bias and controls implemented to adjust for confounding).

Each study was scored on all five categories on the level of risk distinguishing between: low risk, high risk, unclear risk, or mixed risk of bias [26]. Overall risk of bias was determined for each study by its total count of high risk and/or unclear risk of bias scores, and half of the total count of mixed risk of bias instances across categories. Relative risk of bias (low relative risk vs. high relative risk) was determined for each study by comparing each study’s overall risk of bias score with the median overall risk of bias score (below vs. above the median, respectively).

## Results

**Fig. 1:**
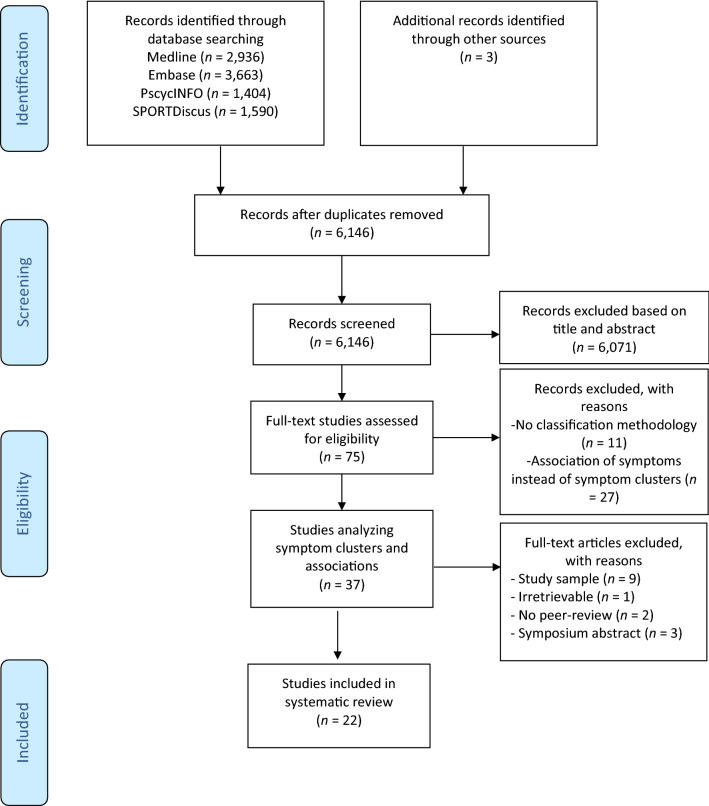
Preferred reporting items for systematic reviews and meta-analyses’ flowchart for study search and selection

A Preferred Reporting Items for Systematic Reviews and Meta-analysis (PRISMA) flow diagram of the study search and selection is displayed in Fig. 1. After deduplication of the 9,593 retrieved records, 6,146 studies remained for further selection. After further assessment of 75 full texts, a total of 22 studies were included for the current systematic review. An overview of study characteristics is provided in Table 1 and a detailed description of risk of bias is displayed in Online Resource 3.

**Table 1:** Summary of characteristics of included studies

Study	Design	SRC Sample (age group, sex size)	Assessment time(s)	Assessment tool	Method of Analysis	RROB
Symptom clusters						
Kontos et al. 2012 [27]	Prospective cohort study	Adolescents (NFS), 67% male (*n* = 1438)	Mean of 2.6 (range 1–7) days post-injury	PCSS-22	EFA	Low
Heyer et al. 2017 [28]	Prospective and retrospective cohort study	Adolescents (NFS), 60% male (*n* = 510)	At time of injury and mean of 9.7 (range 1–30) days post-injury	Symptom questionnaire	PCA	Low
Joyce et al. 2015 [29]	Prospective cohort study	Children–adults (6–22 years), 67% male (*n* = 402)	Mean of 21 days post-injury	PCSS-19	EFA, CFA	Low
Churchill et al. 2017 [30]	Prospective cohort study	Adolescents and adults (< 23 years), 46% male (*n* = 35)	Mean of 3.6 (range = 1–7) days post-injury	SCAT3 (SAC) and MRI	Independent *t* tests	High
Lau et al. 2011 [31]	Prospective cohort study	Adolescents (NFS), 100% male (*n* = 108)	Median of 2 (range 2–3) days post-injury	ImPACT (PCSS-22)	DFA	High
Maruta et al. 2018–2 [32]	Prospective cohort study	Adolescents and adults (NFS), 54% male (*n* = 89)	Mean of 5.8 (SD = 3.5) days post-injury	Modified RPQ	Binomial test	High
Symptom clusters and clinical outcomes						
Heyer et al. 2017*						
Howell et al. 2016 [33]	Prospective cohort study	Children (8–12 years) and adolescents (13–18 years), 67% male (*n* = 318)	Median of 12 (IQR = 8.0, 17.0) days post-injury	PCSS-22	Logistic regression	High
Howell et al. 2018 [34]	Prospective cohort study	Children-adults (7–27 years), 61% male (*n* = 689)	Median of 11 (range = 1–27) days post-injury	PCSS-22	Linear regression	Low
Lau et al. 2009 [35]	Prospective cohort study	Adolescents (NFS), 100% male (*n* = 108)	Not specified (throughout recovery)	ImPACT (PCSS-22)	MANOVAs	High
Lau et al. 2011*						
Lau et al. 2012 [36]	Prospective cohort study	Adolescents (NFS), 100% male (*n* = 108)	Median of 1.91 (short-recovery (≤ 14 days) group) and 2.6 (long (≥ 14 days) recovery group) days post-injury	PCSS-22	ROC curve analysis	High
Sufrinko et al. 2017 [37]	Prospective cohort study	Adolescents (mean 15y, SD = 1.9), 74% male (*n* = 69)	Within 7 days post-injury	PCSS-22	Multinomial logistic regression	Low
Guty et al. 2018 [38]	Prospective cohort study	Adolescents and adults (17–22 years), 82% male (*n* = 122)	Mean of 5.67 (SD = 5.56) days post-injury	ImPACT (PCSS-22)	Independent *t* tests	High
Teel et al. 2017 [39]	Prospective cohort study	Adolescents and adults (NFS), ND (*n* = 375)	At time of injury, 3 h, 1, 2, 3, 5, 7 and 90 days post-injury	GSC, BESS and SAC	GLMM	Low
Brett et al. 2018 [40]	Retrospective cohort study	Adolescents (NFS), 74% male (*n* = 237)	24 h post-injury	SCAT3 (SAC)	Two-step SEM	High
Cohen et al. 2018 [41]	Retrospective cohort study	Adolescents (13–20 years), 70% male (*n* = 72)	Within 1 week post-injury (symptoms), 2–4 weeks post-injury (VOMS and symptom severity)	PCSS-22, VOMS	Linear multiple regression	High
Maruta et al. 2018–1 [42]	Prospective cohort study	Adolescents and adults (14–22 years), 52% male (*n* = 29)	Mean of 5.3 (SD = 3.3) days post-injury	BISQ and eye-tracker	Pearson's correlations	High
Kontos et al. 2013 [43]	Prospective cohort study	Adolescents (NFS), 100% male (*n* = 138)	1–7 days and 8–14 days post-injury	ImPACT (PCSS-22)	ANOVAs	High
Murdaugh et al. 2018 [44]	Prospective cohort study	Children and adolescents (8–18 years), 67% male (*n* = 528)	Within 7 and after 21 days post-injury	ImPACT (PCSS-22)	ANCOVAs	Low
Churchill et al. 2017*						
Paniccia et al. 2018 [45]	Prospective cohort study	Adolescents (13–18 years), 28% male (*n* = 29)	Throughout recovery (weekly follow-up and 1,3 and 6 months post-symptom resolution)	PCSI and 24-h heart rate recording	GLMM	Low
Kontos et al. 2016 [46]	Prospective cohort study	Adolescents and adults (14–29 years), 54% male (*n* = 37)	1– 4 weeks post-injury	PCSS-22, EEG and Auditory Go–NoGo task	ANOVAs	High
Mihalik et al. 2013 [47]	Prospective cohort study	Adolescents and adults (12–25 years), 81% male (*n* = 296)	At time of injury, after the event, 1, 2, 3, 5, 7 and 90 days	GSC, BESS and SAC	GLMM	Low
Sufrinko et al. 2018 [48]	Prospective cohort study	Adolescents (12–18 years), 67% male (*n* = 135)	Within 2 weeks post-injury	ImPACT (PCSS-22) and VOMS	MANOVAs	High

*ANOVAs*, analyses of variance; *ANCOVAs*, analyses of covariance; *BESS*, Balance Error Scoring System; *BISQ*, Brain Injury Screening Questionnaire; *CFA*, Confirmatory Factor Analysis; *DFA*, Discriminant Function Analysis; *EFA*, Exploratory factor analysis; *GLMM*, Generalized linear mixed model; *GSC*, Graded Symptom Checklist; *ImPACT*, Immediate Post-Concussion Assessment and Cognitive Testing; *MANOVAs*, multivariate analyses of variance; *MRI*, magnetic resonance imaging; *ND*, no data; *NFS*, not further specified; *PCA*, Principal Component Analysis; *PCSS*, post-concussion symptom scale; *RPQ*, Rivermead Post-concussion Symptoms Questionnaire; *RROB*, relative risk of bias; *SAC*, Standardized Assessment of Concussion; *SCAT3*, Sport Concussion Assessment Tool 3; *SD*, standard deviation; *SEM*, Structural Equations Modelling; *VOMS*, Vestibular–Ocular-Motor Screening

^*^Same study as mentioned above

### Symptom Clusters

A total of six studies identified clusters of symptom presentations in athletes with SRC by an explorative analysis (*k* = 2), an explorative and a confirmative analysis (*k* = 1), and a supportive analysis (*k* = 3). All symptom clusters identified by these studies were arbitrarily labeled by the reporting authors based on the symptom loadings on each cluster. An overview of these studies and their main findings is provided in Online Resource 4.

#### Explorative Evidence

Kontos et al. 2012 investigated symptom clustering in a large independent sample of concussed high school (*n* = 944) and collegiate (*n* = 494) athletes within 1 week post-injury [27]. The authors identified four symptom clusters, which consisted of a cognitive–migraine–fatigue cluster, an affective cluster, a somatic cluster, and a sleep–arousal cluster. High school athletes reported lower symptom scores on the sleep–arousal cluster than collegiate athletes, suggesting that an older age may be associated with a higher risk of sleep–arousal symptoms after SRC. Likewise, female athletes reported higher symptom scores on the affective symptom cluster than male athletes, suggesting that female athletes may be at higher risk of affective symptoms after SRC. Moreover, the cognitive–migraine–fatigue cluster showed high cross-loadings on all other clusters, suggesting that this cluster may reflect a primary global cluster emerging within the first week post-injury along with secondary and more specific affective, somatic, and sleep–arousal symptom clusters. This study had low relative risk of bias.

Heyer et al. 2017 investigated symptom clustering in a pediatric population with SRC (*n* = 510; 28). The authors found seven distinct clusters, including a dizziness–fogginess cluster, emotional cluster, cephalic cluster, drowsiness cluster, somatic cluster, arousal-stimulation cluster, and vomiting cluster emerging both at the day of concussion injury and at the day of clinical evaluation (M = 9.7 ± 7.8 days post-injury). This study had low relative risk of bias.

#### Explorative and Confirmative Evidence

Joyce et al. 2015 investigated symptom clusters using a large sample of pediatric patients with SRC (*n* = 420) at an average of 21 days post-injury [29]. The authors identified and confirmed three symptom clusters, consisting of a neurocognitive cluster, somatic cluster, and emotional cluster. This study had low relative risk of bias.

#### Supportive Evidence

Churchill et al. 2017 investigated a somatic cluster, cognitive cluster, sleep/fatigue cluster and an emotional cluster in 35 university athletes with a concussion and 35 matched controls at an average of 3.6 ± 3.5 days post-injury [30]. The authors found that a somatic cluster and a cognitive cluster were significantly increased at seven days post-injury relative to baseline measurements as well as symptom reports in a control group. This study had high relative risk of bias.

A study by Lau et al. 2011 investigated a migraine cluster, sleep cluster, cognitive cluster, and neuropsychiatric cluster, as determined by a previously published factor analysis [49], in male high school football athletes (*n* = 108) with SRC within 2–3 days post-injury [31]. The authors confirmed the existence of a migraine cluster, sleep cluster, cognitive cluster, and neuropsychiatric cluster. This study had high relative risk of bias.

A study by Maruta et al. (2018) examined the prevalence of a cognitive–fatigue cluster, a vestibular cluster, an oculomotor cluster, an anxiety/mood cluster, and a migraine cluster, derived through clinical and anecdotal evidence, in 89 athletes at baseline and within 2 weeks of concussion injury [32]. They found that all clusters were more frequently reported by concussed athletes as compared to their baseline measurement. The cognitive–fatigue symptom cluster was most prevalently reported by concussed athletes. This study had high relative risk of bias.

#### Systematic Aggregation of the Clustering Literature

Taken together, the described findings provide convincing evidence for the existence of clusters of SRC symptoms. To provide a systematic aggregation of the clustering literature, we performed MCA to elucidate the most consistent clustering of symptoms across studies. MCA revealed meta-analytic evidence for the existence of five SRC symptom clusters (Table 2). Based on the most influential set of symptoms in each cluster, we identified a *migraine* cluster (i.e. headache, sensitivity to light, sensitivity to noise, nausea), a *cognitive–emotional* cluster (i.e. difficulty remembering, difficulty concentrating, fogginess, feeling more emotional, irritability, feeling slowed down, sadness, nervousness), a *sleep–emotional* cluster (i.e. trouble falling asleep, sleeping less, feeling more emotional, irritability, sleeping more, sadness, nervousness), a *neurological* cluster (blurred vision, vomiting, neck pain, pressure in head, visual problems, double vision), and an *undefined feelings* cluster (“don’t feel right”, confusion).

**Table 2:** MCA-identified SRC symptom clusters

Clusters	Symptoms	Eta-squared
Migraine	Headache	0.748
	Sensitivity to light	0.748
	Sensitivity to noise	0.748
	Nausea	0.644
Cognitive–emotional	Difficulty concentrating	0.578
	Difficulty remembering	0.578
	Fogginess	0.564
	Feeling more emotional	0.521
	Irritability	0.414
	Feeling slowed down	0.379
	Sadness	0.360
	Nervousness	0.360
Sleep–emotional	Trouble falling asleep	0.505
	Sleeping less	0.402
	Feeling more emotional	0.357
	Irritability	0.305
	Sleeping more	0.235
	Sadness	0.234
	Nervousness	0.234
Neurological	Blurred vision	0.550
	Vomiting	0.387
	Neck pain	0.384
	Pressure in head	0.361
	Visual problems	0.251
	Double vision	0.180
Undefined feelings	"Don't feel right"	0.575
	Confusion	0.575

### Symptom Clusters and Clinical Outcomes

A total of 19 studies investigated associations between SRC symptom clusters and clinical outcome variables. These clusters were matched to the clusters as identified by MCA according to the overlap in symptoms that were captured in each cluster (see Online Resource 5). We discuss the clinical relevance of those clusters that mapped to an MCA cluster, showing a minimum overlap of 50% of symptoms (if the studied cluster contained ≥ 50% of symptoms in one of the MCA clusters). An overview of the studies and their main findings is provided in Table 3. If available, standardized effect sizes were reported.

**Table 3:** SRC symptom clusters associated with clinical outcomes

Cluster	Study	Clinical outcome	Effect size
Migraine	Heyer et al. 2017 [28]	Prolonged symptom duration	HR = 1. 34
	Howell et al. 2016 [33]	Prolonged symptom duration	NA
	Howell et al. 2018 [34]	Prolonged symptom duration	NA
	Kontos et al. 2013 [43]	Prolonged recovery (> 21 days)	*d* = 0.52 (compared to headache only) and *d* = 1.10, (compared to no headache)
		Visual memory impairment, verbal memory impairment and slower reaction times	*d* = 0.50 and *d* = 0.34 (compared to headache only and no headache), *d* = 0.70 (compared to headache only) and *d* = 0.54 and *d* = 0.72 (compared to headache only and no headache)
	Mihalik et al. 2013 [47]	Greater total symptom severity scores	NA
	Guty et al. 2018 [38]	Lower neurocognitive scores, lower memory scores and impairment, worse attention/processing speed performance and impairment	*d* = 0.60, d = 0.64, d = 0.54
	Teel et al. 2017 [39]	Balance deficits	NA
	Sufrinko et al. 2018 [48]	Impaired verbal memory, visual memory, visual motor speed and slower reaction time	*d* = 0.55, *d* = 0.70, *d* = 0.55, *d* = 0.40
		More symptoms on smooth pursuits, horizontal saccades, vertical saccades, horizontal and vertical VOR, and VMS	*d* = 1.25, *d* = 1.19, *d* = 1.03, *d* = 1.06, *d* = 1.03, *d* = 1.00
	Churchill et al. 2017 [30]	Higher CBF (compared to cognitive–emotional cluster)	NA
	Kontos et al. 2016 [46]	Lower BNA, decreased N1 amplitude and latency of N1 peak, longer ERP N1 latencies Medial–Frontal, greater total symptom severity scores	*d* = 1.02
Cognitive–emotional	Heyer et al. 2017 [28]	Prolonged symptom duration	HR = 1.20–1.23
	Howell et al. 2018 [34]	Prolonged symptom duration	NA
	Lau et al. 2009 [35]	Complex recovery	*d* = 0.47
	Lau et al. 2012 [36]	Prolonged recovery	NA
	Teel et al. 2017 [39]	Cognitive and balance deficits	NA
	Cohen et al. 2018 [41]	Greater total symptom severity scores	NA
	Churchill et al. 2017 [30]	Lower CBF (compared to migraine cluster)	NA
	Paniccia et al. 2018 [45]	Increased pNN50, HF, Hfnu	NA
Sleep–emotional	Heyer et al. 2017 [28]	Prolonged symptom duration	HR = 1.23
	Lau et al. 2009 [35]	Complex recovery	*d* = 0.44
	Sufrinko et al. 2017 [37]	Longer recovery (30–90 dayssleep)	*d* = 0.16
	Kontos et al. 2013 [43]	Less sleep quantity	*r* = -.14
	Guty et al. 2018 [38]	Lower memory scores and impairment	*d* = 0.53
	Teel et al. 2017 [39]	Balance deficits	NA
	Cohen et al. 2018 [41]	Greater total symptom severity scores	NA
	Murdaugh et al. 2018 [44]	Less sleep quantity	NA
	Paniccia et al. 2018 [45]	Increased HF and Hfnu	NA
Neurological	Cohen et al. 2018 [41]	More symptoms during smooth pursuit, horizontal & vertical saccades, vertical saccades, horizontal and vertical VOR, VMS, and NPC	NA

*BNA*, brain network activation; *CBF*, cerebral blood flow; *d*, Cohen’s d; *ERP*, event-related potential; *GSC*, graded symptom checklist; *HF*, high frequency; *Hfnu*, High-frequency normalized units; *HR*, log hazard ratio; *NA*, not available; *NPC*, near point of convergence; *r*, Pearson correlations; *pNN50*, number of pairs of successive RRs that differ by more than 50 ms, divided by total number of RRs. *VMS*, visual motion sensitivity; *VOR*, vestibular–ocular reflex

#### Migraine Cluster

A total of ten (24%) of reported clusters mapped most closely to the *migraine* cluster. Clusters mapping to the *migraine* cluster were found to be associated with prolonged recovery/symptom duration (*k* = 4), cognitive deficits (*k* = 3), neuroimaging parameters (*k* = 2), balance deficits (*k* = 1), greater total symptom severity scores (*k* = 1), and provoked vestibular–ocular-motor screening symptoms (k = 1).

#### Cognitive–Emotional Cluster

A total of eight (19%) of reported clusters had the highest match to the *cognitive–emotional* cluster. Clusters mapping to the *cognitive–emotional* cluster were found to be associated with prolonged recovery/symptom duration (*k* = 4), cognitive and balance deficits (*k* = 1), neuroimaging parameters (*k* = 1), greater total symptom severity scores (*k* = 1), and heart rate recordings (*k* = 1).

#### Sleep–Emotional Cluster

Among the reported clusters, nine (21%) mapped to the *sleep–emotional* cluster. These clusters were found to be associated with prolonged recovery/symptom duration (*k* = 3), lower sleep quantity (*k* = 2), cognitive deficits (*k* = 1), balance deficits (*k* = 1), greater total symptom severity scores (*k* = 1), and heart rate recordings (*k* = 1).

#### Neurological Cluster

One cluster (2%) with the highest match to the *neurological* cluster was found to be associated with provoked vestibular–ocular-motor screening symptoms (*k* = 1).

#### Undefined Feelings Cluster

None of the reported clusters in the literature had the highest match to the *undefined feelings* cluster. Therefore, we found no evidence for a relation between this cluster and clinical outcome.

#### Summary

Clusters reported in the literature mapped most frequently to the *migraine* cluster (24%), followed by the *sleep–emotional* cluster (21%), the *cognitive–emotional* cluster (19%), and the *neurologica*l cluster (2%), while none of the remaining clusters in the literature had the highest match to the *undefined feelings* cluster (0%).

The strength of the relation between symptom clusters and clinical outcomes ranged between small and large, with 28% of the relations showing small effect sizes (relations between the *migraine*, *cognitive–emotional*, *sleep–emotional* cluster and clinical outcomes), 41% of the relations showing moderate effect sizes (relations between the *migraine*, *cognitive–emotional*, *sleep–emotional* cluster and clinical outcomes) and 28% of the relations showing large effect sizes (relations between the *migraine* cluster and clinical outcomes). These findings suggest that the *migraine* cluster is most strongly associated with clinical outcome, while no evidence was found for associations between the *undefined feelings* cluster and clinical outcome. Relations between symptom clusters and clinical outcomes were also consistently observed by studies with low relative risk of bias, indicating that study quality did not account for the observed associations between clusters and clinical outcome measures. Together, these findings suggest that SRC symptom clusters are associated with clinical outcomes, while it remains unclear if and which specific SRC symptom clusters are associated with impairments in particular functional domains.

## Discussion

This systematic review is the first to (i) systematically evaluate and integrate all available evidence on the classification of SRC symptoms into clusters, (ii) aggregate the available evidence using a meta-analytic approach, and (iii) assess the relation between SRC symptom clusters and clinical outcome. Findings derived from 22 studies representing 5592 athletes with SRC provide strong and consistent evidence for the existence of SRC symptom clusters and relevance of these clusters for clinical outcome. The study findings contribute to our understanding of the distinct heterogeneity of SRC and strongly support a transition from a unitary approach to SRC clinical management towards individualized and targeted SRC prognosis, clinical management, and intervention strategies.

Studies that performed data-driven exploration of symptom clusters provided strong and consistent evidence for clustering of SRC symptoms. The clustering of symptoms was subject to variability between studies, both in the number of clusters (ranging between 2 and 7 cluster solutions) and the symptoms encompassed in the clusters (i.e. the specific symptom clustering together). These variable results may be explained by differences in the methodology of studies (e.g. instrument used for symptom assessment, study sample characteristics, time post-injury). In an attempt to integrate the available evidence and provide an overarching interpretation of symptom clustering across studies, we used MCA to quantitatively investigate the correspondence between the clusters reported in the literature. The results revealed meta-analytic evidence for the most consistently reported clusters, which were identified as a *migraine* cluster, a *cognitive–emotional* cluster, a *sleep–emotional* cluster, a *neurological* cluster, and an *undefined feelings* cluster. These findings provide convincing evidence for the existence of (at least) five subtypes in SRC symptomatology.

The observed evidence for the existence of SRC subtypes is in line with the existing literature, such as the practice-based model for delineation of SRC symptoms as proposed by Collins and colleagues [22] that was recently revised in an expert consensus-driven description of concussion subtypes [50]. The current study used data-driven classification to empirically confirm the segregation of SRC symptoms into symptom clusters. With regard to specific subtypes, this study directly confirms the existence of distinct symptom clusters relating to migraine, cognitive, and emotional symptoms [50]. However, in contrast to consensus-driven definitions, we found cognitive symptoms to cluster with emotional symptoms (i.e. cognitive–emotional symptom cluster), sleep symptoms to cluster with emotional symptoms (i.e. sleep–emotional symptom cluster), and ocular symptoms to cluster with vestibular and cervical symptoms (i.e. neurological symptom cluster). The current study extends the consensus-driven description of subtypes by providing a meta-analytic aggregation of all available evidence on the classification of SRC symptoms into clusters, resulting in an empirical taxonomy of symptom clusters, and by assessing the relation between these symptom clusters and clinical outcomes.

Studies included in the current review also support the idea that there are meaningful relationships between SRC symptom clusters and clinical outcomes. More specifically, our findings showed that clusters reported in the literature mapped most frequently to the *migraine* cluster. Moreover, the effect sizes for the reported associations between the *migraine* cluster and clinical outcomes were larger compared to the other clusters. Findings also showed that the *sleep–emotional* cluster, the *cognitive–emotional* cluster, and the *neurological* cluster were related to clinical outcome, while no evidence was found for associations between the *undefined feelings* cluster and clinical outcome. These observed associations between SRC symptom clusters and meaningful clinical outcomes further support the idea that subtypes require targeted clinical management and treatment to improve athlete outcomes.

### Limitations of Available Evidence

The current systematic review has some weaknesses, first determined by the limitations of included studies. Most of these studies had limitations that may have influenced the validity of their findings. For example, only 13.6% of included studies performed explorative data-driven classification of SRC symptom clustering, without prior-formulating these symptom clusters based on hypothesis or other research, and as such, analysis bias may have confounded the identified clusters. Nevertheless this study still aimed to provide a systematic aggregation of SRC clustering, by performing MCA to elucidate the most consistent clustering of symptoms across studies. Moreover, all studies included in this systematic review investigated SRC symptom clustering within the typical phase of recovery (within 1 month post-injury). Consequently, little is known about the classification of SRC symptoms into clusters beyond this recovery phase, which is especially relevant for more complex forms of SRC and the development of persistent symptoms after concussion. Finally, only five of the included studies adjusted for covariates, such as age, sex, and post-injury time, in their analysis, which may also be important confounders and, therefore, may have contributed to bias of the results.

### Implications and Recommendations for Future Research

SRC subtyping may facilitate the symptom-targeted treatment approach through identification of the relevant combination of available treatments (e.g. headache treatment, vestibular treatment, psychological treatment, physical therapy, targeted life style interventions) and the development of novel treatments (e.g. pharmacotherapy, active exercise-based interventions; 4,51,52). It remains unclear, however, how SRC subtypes differentially relate to specific functional impairments. Future studies may importantly contribute by mapping symptom clusters to objective deficits in functional outcome domains measures. This requires the use of a broad battery of functional assessments across domains. Based on our findings, the state of the literature warrants systematic assessment of at least headache characteristics, neurocognitive functioning, emotional functioning, neurological functioning, and sleep. In addition, future studies should further investigate the relation between SRC symptom clustering and variables that might be predictive of the emergence of SRC clusters, such as demographic and injury-related variables [27, 28]. Although it remains unknown to what extent subtypes of concussion are specific to SRC, the current evidence for the clinical relevance of SRC subtyping may also be highly relevant for future research in the broader context of (mild) traumatic brain injury. Since symptoms clusters may overlap and/or co-occur, it could also be valuable to investigate the potential existence of patient subtypes that exhibit a comparable configuration of symptom subtypes. Furthermore, more research is needed with regard to the evolution of SRC (subtypes of) symptoms over time, especially beyond the typical course of recovery (> 1 month; 13,14). An innovative approach to investigate the dynamic inter-relationships among persistent SRC symptoms is a network analysis that was recently proposed by Iverson [53]. This network perspective for persistent symptoms posits that a SRC can be viewed as a set of interacting symptoms in which symptoms co-occur, because they are strongly inter-related, activating, and amplifying. Adopting network analysis in future SRC research may improve our understanding of the temporal dynamics of SRC symptoms.

## Conclusions

Systematic and meta-analytic evaluation of the literature provides robust evidence for the existence of SRC symptom subtypes. Meta-analysis of the available literature provides evidence for the existence of at least five SRC clusters, identified as a *migraine* cluster, a *cognitive–emotional* cluster, a *sleep–emotional* cluster, a *neurological* cluster, and an *undefined feelings* cluster. Clusters mapping to the *migraine* cluster were most frequently reported in the literature and were most strongly related to aspects of clinical outcome, while there was also evidence for the clinical relevance of the *cognitive–emotional*, *sleep–emotional*, and *neurological* clusters. Taken together, the state of the literature clearly highlights the clinical relevance of SRC symptom subtyping. The results of this study may pave the way for the transition from a unitary approach to SRC management towards individualized and targeted concussion management and treatment, with the ultimate aim to optimize the recovery of SRC and prevent the development of persistent symptoms after concussion.

## Electronic supplementary material

Below is the link to the electronic supplementary material.

Supplementary file1 (DOCX 24 kb)

Supplementary file2 (DOCX 174 kb)

Supplementary file3 (DOCX 46 kb)

Supplementary file4 (DOCX 25 kb)

Supplementary file5 (DOCX 42 kb)

## Data Availability

The data that support the findings of this study are available from the corresponding author upon reasonable request.
