# Supplementary material for: Subtypes of Sport-Related Concussion: a Systematic Review and Meta-cluster Analysis
Source: Sports Med. 2020 Jul 27;50(10):1829–42. doi: 10.1007/s40279-020-01321-9 (PMC7497426; doi:10.1007/s40279-020-01321-9)
Supplement: Supplementary file 1 — Supplementary file1 (DOCX 24 kb) [file 40279_2020_1321_MOESM1_ESM.docx]

Subtypes of Sport-Related Concussion: a Systematic Review and Meta-Cluster Analysis

Sports Medicine

S Langdon MSc*^#^, M Königs PhD*, E A M C Adang BSc*, E Goedhart MD⁺, J Oosterlaan, PhD*

**Emma Children’s Hospital, Amsterdam University Medical Centers (location Academic Medical Center), Meibergdreef 9, 1105 AZ Amsterdam, The Netherlands.*

*^#^Corresponding author, e-mail address: s.langdon@amsterdamumc.nl*

⁺*Sport Medical Centre,* *Royal Dutch Football Association (KNVB), Woudenbergseweg 56-58, 3707 HX Zeist, The Netherlands.*

# **Online Resource 1 – Search Queries**

|  | **Searches** | **Results** | **After deduplication** | **19 Dec additional records** | **Total** |
| --- | --- | --- | --- | --- | --- |
| **#** | **Ovid MEDLINE(R) Epub Ahead of Print, In-Process & Other Non-Indexed Citations, Ovid MEDLINE(R) Daily and Ovid MEDLINE(R) <1946 to Present> Search date: 15 March 2018** |  |  |  |  |
| 1 | exp brain concussion/ | 6977 |  |  |  |
| 2 | (concuss* or contrecoup or contre coup or postconcuss*).ab,kf,ti. | 7891 |  |  |  |
| 3 | 1 or 2 [concussion] | 10569 |  |  |  |
| 4 | exp sports/ or exp sports medicine/ or psychology, sports/ | 166298 |  |  |  |
| 5 | (athlet* or sport* or gymnast*).ab,jw,kf,ti. | 149748 |  |  |  |
| 6 | ((physical or recreational) adj3 activit*).ab,kf,ti. | 95006 |  |  |  |
| 7 | or/4-6 [sport- or activity related] | 325665 |  |  |  |
| 8 | guideline.pt. | 15927 |  |  |  |
| 9 | (statement or guideline? or protocol? or consensus).mp. | 1000873 |  |  |  |
| 10 | (assess* or evaluat* or scale or inventor* or questionn* or survey? or (symptom? adj10 measure*)).mp. | 6044030 |  |  |  |
| 11 | or/8-10 [guidelines \| symptoms measurement] | 6614651 |  |  |  |
| 12 | and/3,7,11 | 2540 |  |  |  |
| 13 | (ncaa dod or ncaa concussion or (ncaa adj5 (study or trial))).ab,kf,ti. | 33 |  |  |  |
| 14 | (cisg or "concussion in sport group").ab,kf,ti. | 24 |  |  |  |
| 15 | 13 or 14 [relevant studies] | 57 |  |  |  |
| 16 | 12 or 15 | 2565 |  |  |  |
| 17 | remove duplicates from 16 | 2561 | **2289** | **375** | **2664** |
|  |  |  |  |  |  |
|  | **Embase Classic+Embase <1947 to 2018 March 14> Search date 15 March 2018** |  |  |  |  |
| 1 | brain concussion/ or postconcussion syndrome.mp. [mp=title, abstract, heading word, drug trade name, original title, device manufacturer, drug manufacturer, device trade name, keyword, floating subheading word] | 6981 |  |  |  |
| 2 | (concuss* or contrecoup or contre coup or postconcuss*).ab,kw,ti. | 11113 |  |  |  |
| 3 | 1 or 2 [concussion] | 13367 |  |  |  |
| 4 | exp *sport/ or sports medicine/ or sports psychology/ | 80756 |  |  |  |
| 5 | (athlet* or sport* or gymnast*).ab,jx,kw,ti. | 181037 |  |  |  |
| 6 | ((physical or recreational) adj3 activit*).ab,kw,ti. | 131511 |  |  |  |
| 7 | or/4-6 [sport- or activity related] | 328152 |  |  |  |
| 8 | consensus development/ or *practice guideline/ | 78420 |  |  |  |
| 9 | (statement or guideline? or protocol? or consensus).mp. | 1324650 |  |  |  |
| 10 | (assess* or evaluat* or scale or inventor* or questionn* or survey? or (symptom? adj10 measure*)).mp. | 8894531 |  |  |  |
| 11 | or/8-10 [guidelines \| symptoms measurement] | 9566898 |  |  |  |
| 12 | and/3,7,11 | 3473 |  |  |  |
| 13 | (ncaa dod or ncaa concussion or (ncaa adj5 (study or trial))).ab,kw,ti. | 68 |  |  |  |
| 14 | (cisg or "concussion in sport group").ab,kw,ti. | 24 |  |  |  |
| 15 | 13 or 14 [relevant studies] | 92 |  |  |  |
| 16 | 12 or 15 | 3530 |  |  |  |
| 17 | remove duplicates from 16 | 3423 | **1383** | **240** | **1623** |
|  |  |  |  |  |  |
|  | **Ovid PsycINFO <1806 to March Week 1 2018> Search date: 15 March 2018** |  |  |  |  |
| 1 | brain concussion/ | 1642 |  |  |  |
| 2 | (concuss* or contrecoup or contre coup or postconcuss*).ab,id,ti. | 3090 |  |  |  |
| 3 | 1 or 2 [concussion] | 3118 |  |  |  |
| 4 | exp sports/ or sports medicine/ or sport psychology/ | 25832 |  |  |  |
| 5 | (athlet* or sport* or gymnast*).ab,jx,id,ti. | 44128 |  |  |  |
| 6 | ((physical or recreational) adj3 activit*).ab,id,ti. | 32456 |  |  |  |
| 7 | or/4-6 [sport- or activity related] | 76437 |  |  |  |
| 8 | 3 and 7 | 1265 |  |  |  |
| 9 | (ncaa dod or ncaa concussion or (ncaa adj5 (study or trial))).ab,id,ti. | 52 |  |  |  |
| 10 | (cisg or "concussion in sport group").ab,id,ti. | 2 |  |  |  |
| 11 | 9 or 10 [relevant studies] | 54 |  |  |  |
| 12 | 8 or 11 | 1314 | **773** | **90** | **863** |
|  |  |  |  |  |  |
|  | **Ebscohost SPORTDiscus Search date: 15 March 2018** |  |  |  |  |
| 1 | DE "BRAIN - Concussion" OR DE "POSTCONCUSSION syndrome" | 147 |  |  |  |
| 2 | AB (concuss* or contrecoup or contre coup or postconcuss*) OR KW (concuss* or contrecoup or contre coup or postconcuss*) OR TI (concuss* or contrecoup or contre coup or postconcuss*) | 3703 |  |  |  |
| 3 | S1 OR S2 | 3709 |  |  |  |
| 4 | AB (statement or guideline? or protocol? or consensus) OR KW (statement or guideline? or protocol? or consensus) OR TI (statement or guideline? or protocol? or consensus) | 34777 |  |  |  |
| 5 | AB (assess* or evaluat* or scale or inventor* or questionn* or survey? or (symptom? NEAR/9 measure*)) OR KW (assess* or evaluat* or scale or inventor* or questionn* or survey? or (symptom? NEAR/9 measure*)) OR TI (assess* or evaluat* or scale or inventor* or questionn* or survey? or (symptom? NEAR/9 measure*)) | 263234 |  |  |  |
| 6 | S4 OR S5 | 288262 |  |  |  |
| 7 | S3 AND S6 | 1400 |  |  |  |
| 8 | DE "SPORT Concussion Assessment Tool" | 8 |  |  |  |
| 9 | AB (ncaa dod or ncaa concussion or (ncaa NEAR/4 (study or trial))) OR KW (ncaa dod or ncaa concussion or (ncaa NEAR/4 (study or trial))) OR TI (ncaa dod or ncaa concussion or (ncaa NEAR/4 (study or trial))) | 44 |  |  |  |
| 10 | AB (cisg or "concussion in sport group") OR KW (cisg or "concussion in sport group") OR TI (cisg or "concussion in sport group") | 15 |  |  |  |
| 11 | S7 OR S8 OR S9 OR S10 | 1439 | **842** | **151** | **993** |
