# Supplementary material for: Subtypes of Sport-Related Concussion: a Systematic Review and Meta-cluster Analysis
Source: Sports Med. 2020 Jul 27;50(10):1829–42. doi: 10.1007/s40279-020-01321-9 (PMC7497426; doi:10.1007/s40279-020-01321-9)
Supplement: Supplementary file 2 — Supplementary file2 (DOCX 174 kb) [file 40279_2020_1321_MOESM2_ESM.docx]

Subtypes of Sport-Related Concussion: a Systematic Review and Meta-Cluster Analysis

Sports Medicine

S Langdon MSc*^#^, M Königs PhD*, E A M C Adang BSc*, E Goedhart MD⁺, J Oosterlaan, PhD*

**Emma Children’s Hospital, Amsterdam University Medical Centers (location Academic Medical Center), Meibergdreef 9, 1105 AZ Amsterdam, The Netherlands.*

*^#^Corresponding author, e-mail address: s.langdon@amsterdamumc.nl*

⁺*Sport Medical Centre,* *Royal Dutch Football Association (KNVB), Woudenbergseweg 56-58, 3707 HX Zeist, The Netherlands.*

# **Online Resource 2 – Scree Plots**

##
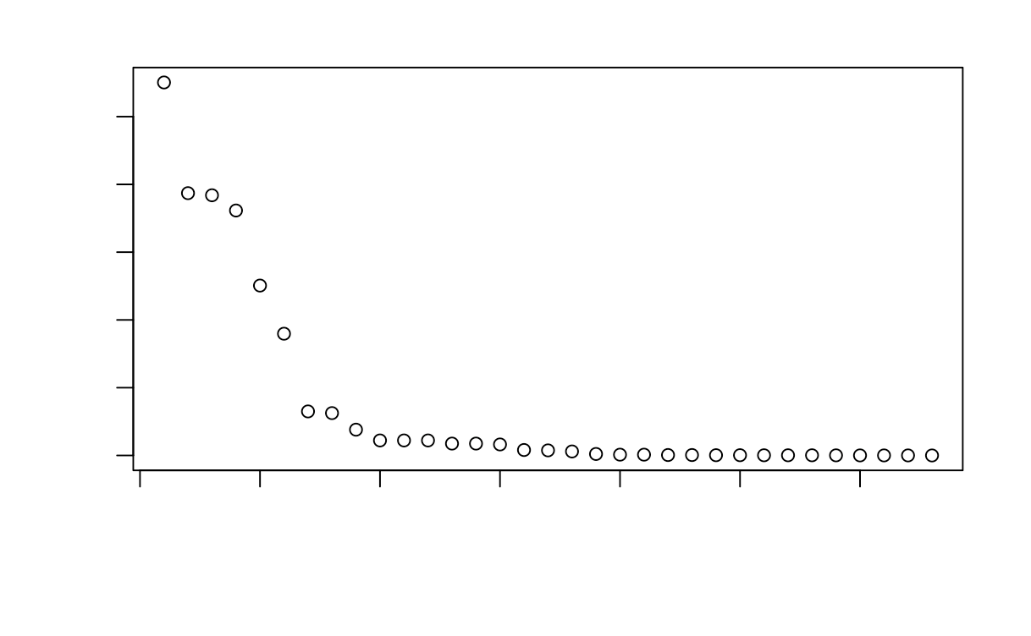

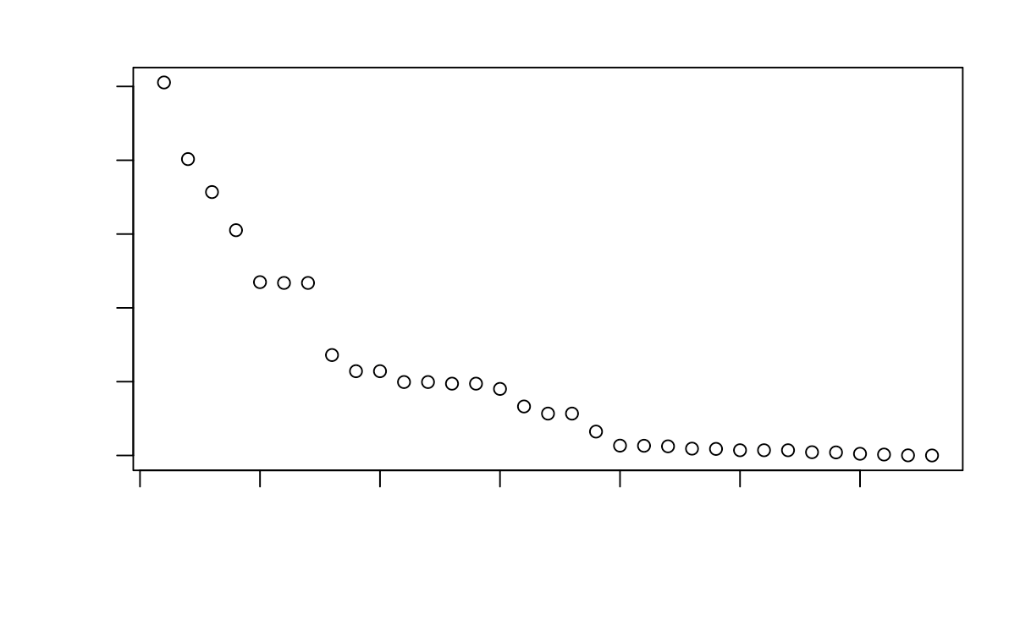

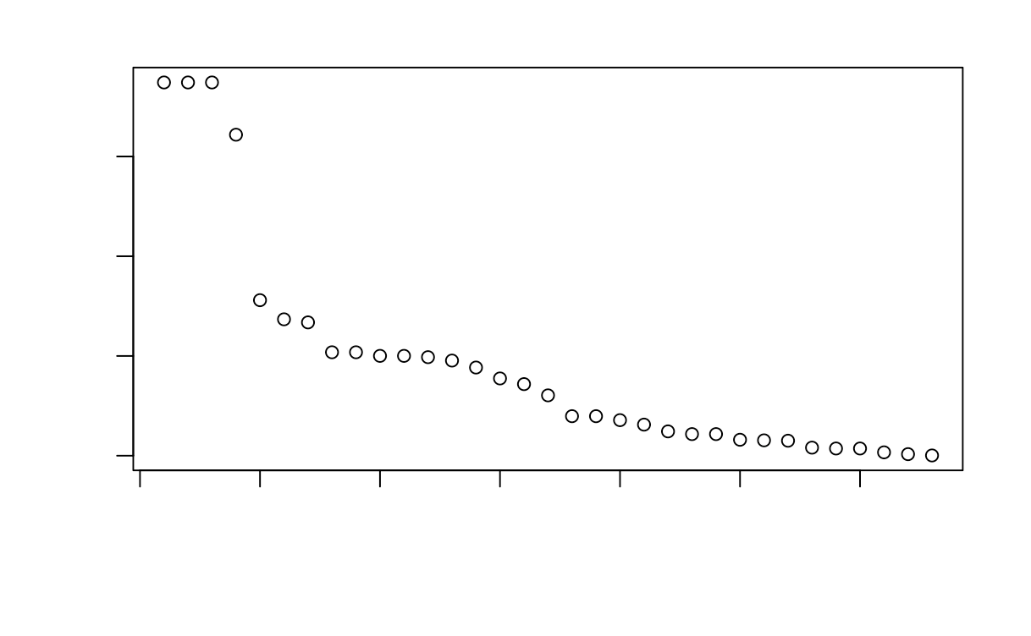

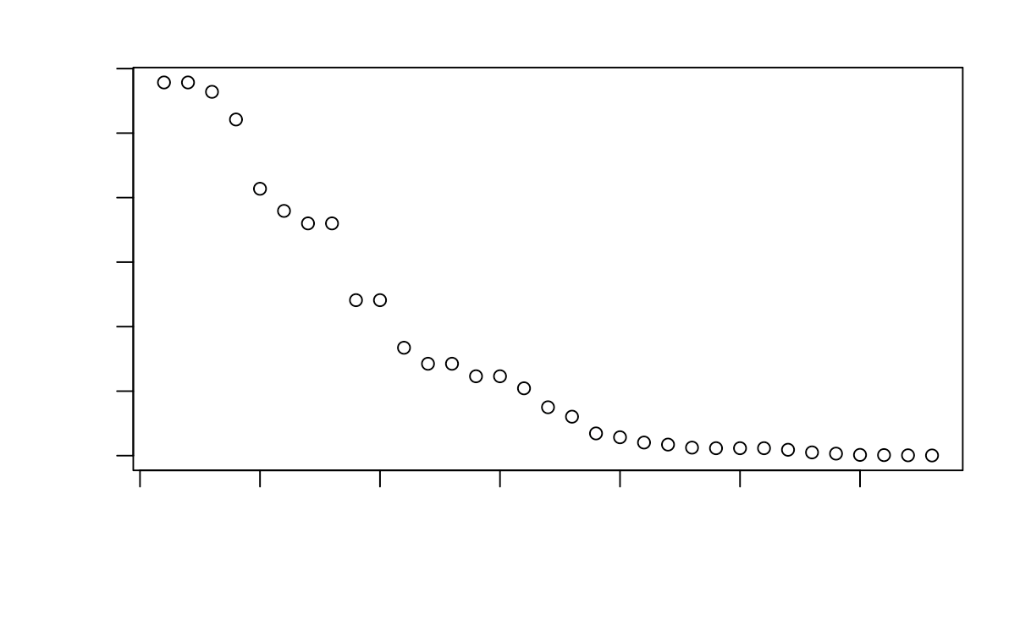


*Sleep-emotional* cluster

*Cognitive-emotional* cluster

*Migraine* cluster

*Neurological* cluster

Ƞ^2^

Ƞ^2^

Symptoms

Symptoms

Symptoms

Symptoms

Ƞ^2^

Symptoms

Ƞ^2^

Symptoms


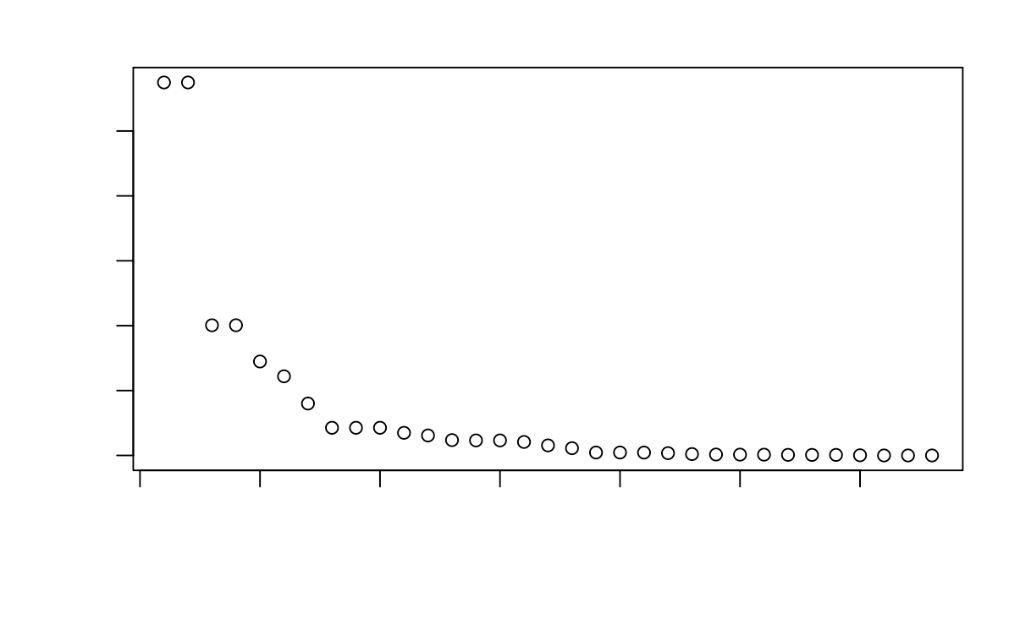


*Undefined feelings* cluster

Symptoms

Ƞ^2^

= boundary of a set of symptoms with the strongest contribution to a cluster in terms of effect size (ƞ^2^)
