# Supplementary material for: Subtypes of Sport-Related Concussion: a Systematic Review and Meta-cluster Analysis
Source: Sports Med. 2020 Jul 27;50(10):1829–42. doi: 10.1007/s40279-020-01321-9 (PMC7497426; doi:10.1007/s40279-020-01321-9)
Supplement: Supplementary file 3 — Supplementary file3 (DOCX 46 kb) [file 40279_2020_1321_MOESM3_ESM.docx]

Subtypes of Sport-Related Concussion: a Systematic Review and Meta-Cluster Analysis

Sports Medicine

S Langdon MSc*^#^, M Königs PhD*, E A M C Adang BSc*, E Goedhart MD⁺, J Oosterlaan, PhD*

**Emma Children’s Hospital, Amsterdam University Medical Centers (location Academic Medical Center), Meibergdreef 9, 1105 AZ Amsterdam, The Netherlands.*

*^#^Corresponding author, e-mail address: s.langdon@amsterdamumc.nl*

⁺*Sport Medical Centre,* *Royal Dutch Football Association (KNVB), Woudenbergseweg 56-58, 3707 HX Zeist, The Netherlands.*

# **Online Resource 3 – Risk of bias assessment**

|  | Selection Bias | |  | Detection Bias |  | Performance Bias | |  | Follow-up Bias | |  | Other | |
| --- | --- | --- | --- | --- | --- | --- | --- | --- | --- | --- | --- | --- | --- |
| Study | Representative patient group | Adequate case definition |  | Outcome Assessor Blinding |  | Outcome Patient Blinding | Outcome Objectivity |  | Follow-up | Attrition |  | Analysis Bias | Confounding |
| Kontos et al. 2012 (21) | Yes | Yes |  | ND |  | ND | Patient-rated |  | No | NA |  | Data driven | No |
|  | Low risk | Low risk |  | Unclear risk |  | Unclear | High risk |  | High risk | - |  | Low risk | Low risk |
| Heyer et al. 2017 (24) | Yes | Yes |  | ND |  | ND | Patient-rated |  | Yes | ND |  | Data driven | ND |
|  | Low risk | Low risk |  | Unclear risk |  | Unclear risk | High risk |  | Low risk | Unclear risk |  | Low risk | Unclear risk |
| Jocye et al. 2015 (25) | Yes | Yes |  | ND |  | ND | Patient-rated |  | No | NA |  | Data driven | No |
|  | Low risk | Low risk |  | Unclear risk |  | Unclear risk | High risk |  | High risk | - |  | Low risk | Low risk |
| Churchill et al. 2017 (26) | Yes | Yes |  | ND |  | ND | Patient-rated and performance based |  | No | NA |  | Hypothesis driven | ND |
|  | Low risk | Low risk |  | Unclear risk |  | Unclear risk | Mixed risk |  | High risk | - |  | High risk | Unclear risk |
| Lau et al. 2011 (45) | Yes | Yes |  | ND |  | ND | Patient-rated and performance based |  | Yes | ND |  | Hypothesis-driven | ND |
|  | Low risk | Low risk |  | Unclear risk |  | Unclear risk | Mixed risk |  | Low risk | Unclear risk |  | High risk | Unclear risk |
| Maruta et al. 2018-2 (28) | Yes | Yes |  | ND |  | ND | Patient-rated |  | No | NA |  | Hypothesis driven | ND |
|  | Low risk | Low risk |  | Unclear risk |  | Unclear risk | High risk |  | High risk | - |  | High risk | Unclear risk |
| Howell et al. 2016 (29) | Yes | Yes |  | ND |  | ND | Patient-rated |  | No | NA |  | Hypothesis driven | ND |
|  | Low risk | Low risk |  | Unclear risk |  | Unclear risk | High risk |  | High risk | - |  | High risk | Unclear risk |
| Howell et al. 2018 (30) | Yes | Yes |  | ND |  | ND | Patient-rated |  | No | NA |  | Hypothesis driven | No |
|  | Low risk | Low risk |  | Unclear risk |  | Unclear risk | High risk |  | High risk | - |  | High risk | Low risk |
| Lau et al. 2009 (31) | Yes | Yes |  | ND |  | ND | Patient-rated and perfomance based |  | Yes | ND |  | Hypothesis-driven | ND |
|  | Low risk | Low risk |  | Unclear risk |  | Unclear risk | Mixed risk |  | Low risk | Unclear risk |  | High risk | Unclear risk |
| Lau et al. 2012 (32) | Yes | Yes |  | ND |  | ND | Patient-rated |  | No | NA |  | Hypothesis-driven | ND |
|  | Low risk | Low risk |  | Unclear risk |  | Unclear risk | High risk |  | High risk | - |  | High risk | Unclear risk |
| Sufrinko et al. 2017 (33) | Yes | Yes |  | ND |  | ND | Patient-rated and performance based |  | Yes | 100% |  | Hypothesis-driven | ND |
|  | Low risk | Low risk |  | Unclear risk |  | Unclear risk | Mixed risk |  | Low risk | Low risk |  | High risk | Unclear risk |
| Guty et al. 2018 (34) | Yes | Yes |  | ND |  | ND | Patient-rated and performance based |  | No | NA |  | Hypothesis-driven | ND |
|  | Low risk | Low risk |  | Unclear risk |  | Unclear risk | Mixed risk |  | High risk | - |  | High risk | Unclear risk |
| Teel et al. 2017 (46) | Yes | Yes |  | ND |  | ND | Patient-rated and performance based |  | Yes | 37-42% |  | Hypothesis driven | ND |
|  | Low risk | Low risk |  | Unclear risk |  | Unclear risk | Mixed risk |  | Low risk | Mixed risk |  | High risk | Unclear risk |
| Brett et al. 2018 (36) | Yes | Yes |  | ND |  | ND | Patient-rated and performance based |  | No | NA |  | Hypothesis driven | ND |
|  | Low risk | Low risk |  | Unclear risk |  | Unclear risk | Mixed risk |  | High risk | - |  | High risk | Unclear risk |
| Cohen et al. 2018 (37) | Yes | Yes |  | ND |  | ND | Patient-rated and performance based |  | No | NA |  | Hypothesis-driven | ND |
|  | Low risk | Low risk |  | Unclear risk |  | Unclear risk | Mixed risk |  | High risk | - |  | High risk | Unclear risk |
| Maruta et al. 2018-1 (38) | Yes | Yes |  | ND |  | ND | Patient-rated and performance based |  | No | NA |  | Hypothesis-driven | ND |
|  | Low risk | Low risk |  | Unclear risk |  | Unclear risk | Mixed risk |  | High risk | - |  | High risk | Unclear risk |
| Kontos et al. 2013 (39) | Yes | Yes |  | ND |  | ND | Patient-rated and performance based |  | Yes | ND |  | Hypothesis driven | ND |
|  | Low risk | Low risk |  | Unclear risk |  | Unclear risk | Mixed risk |  | Low risk | Unclear risk |  | High risk | Unclear risk |
| Murdaugh et al. 2018 (40) | Yes | Yes |  | ND |  | ND | Patient-rated and performance based |  | Yes | 100% |  | Hypothesis-driven | No |
|  | Low risk | Low risk |  | Unclear risk |  | Unclear risk | Mixed risk |  | Low risk | Low risk |  | High risk | Low risk |
| Paniccia et al. 2018 (41) | Yes | Yes |  | ND |  | ND | Patient-rated and performance based |  | Yes | ND |  | Hypothesis-driven | No |
|  | Low risk | Low risk |  | Unclear risk |  | Unclear risk | Mixed risk |  | Low risk | Unclear risk |  | High risk | Low risk |
| Kontos et al. 2016 (47) | Yes | Yes |  | ND |  | ND | Patient-rated and performance based |  | Yes | ND |  | Hypothesis-driven | ND |
|  | Low risk | Low risk |  | Unclear risk |  | Unclear risk | Mixed risk |  | Low risk | Unclear risk |  | High risk | Unclear risk |
| Mihalik et al. 2013 (43) | Yes | Yes |  | ND |  | ND | Patient-rated and performance based |  | Yes | ND |  | Hypothesis driven | No |
|  | Low risk | Low risk |  | Unclear risk |  | Unclear risk | Mixed risk |  | Low risk | Unclear risk |  | High risk | Low risk |
| Sufrinko et al. 2018 (44) | Yes | Yes |  | ND |  | ND | Patient-rated and performance based |  | No | NA |  | Hypothesis-driven | ND |
|  | Low risk | Low risk |  | Unclear risk |  | Unclear risk | Mixed risk |  | High risk | - |  | High risk | Unclear risk |
| Abbreviations: ND, no data; NA, not applicable. | | | | | | | | | | | | | |
