# Supplementary material for: Subtypes of Sport-Related Concussion: a Systematic Review and Meta-cluster Analysis
Source: Sports Med. 2020 Jul 27;50(10):1829–42. doi: 10.1007/s40279-020-01321-9 (PMC7497426; doi:10.1007/s40279-020-01321-9)
Supplement: Supplementary file 4 — Supplementary file4 (DOCX 25 kb) [file 40279_2020_1321_MOESM4_ESM.docx]

Subtypes of Sport-Related Concussion: a Systematic Review and Meta-Cluster Analysis

Sports Medicine

S Langdon MSc*^#^, M Königs PhD*, E A M C Adang BSc*, E Goedhart MD⁺, J Oosterlaan, PhD*

**Emma Children’s Hospital, Amsterdam University Medical Centers (location Academic Medical Center), Meibergdreef 9, 1105 AZ Amsterdam, The Netherlands.*

*^#^Corresponding author, e-mail address: s.langdon@amsterdamumc.nl*

⁺*Sport Medical Centre,* *Royal Dutch Football Association (KNVB), Woudenbergseweg 56-58, 3707 HX Zeist, The Netherlands.*

# **Online Resource 4 – Identified SRC symptoms clusters**

| Study | | Assessment tool | | Post-injury time | | Significant clusters (*symptoms*) | | |  |  |
| --- | --- | --- | --- | --- | --- | --- | --- | --- | --- | --- |
| *2-cluster solution* | |  | |  | |  | |  |  |  |
| Brett et al. 2018 (36) | | SCAT3 | | 24 hours | | - **CMF** (*headache, dizziness, fatigue, drowsiness, sensitivity to light, sensitivity to noise, feeling slowed down, mentally foggy, difficulty remembering, immediate memory, postural instability*) - **Somatic** (*vomiting, numbness*) | | | | |
| Churchill et al. 2017 (26) | | SCAT3 (SAC) | | 1-7 days | | - **Somatic** (*headache, pressure in head, neck pain, nausea/vomiting, dizziness, blurred vision, balance problems, sensitivity to light, sensitivity to noise*) - **Cognitive** ( *feeling slowed down, feeling "in a fog" , "don't feel right", difficulty concentrating, difficulty remembering, confusion*) | | | | |
|  | |  | |  | |  | |  | | |
| *3-cluster solution* | |  | |  | |  | |  | | |
| Joyce et al. 2015 (25) | | PCSS-19 | | Mean of 21 days post-injury | | - **Somatic** (*headache, nausea, sleeping more than usual, sensitivity to light, sensitivity to noise, trouble falling asleep*) - **Neurocognitive** (*balance problems, dizziness, drowsiness, numbness or tingling, difficulty concentrating, feeling as if 'in a fog', difficulty remembering, feeling slowed down*) - **Emotional** (*irritability, nervousness, sadness, more emotional than usual*) | | | | |
|  | |  | |  | |  | |  | | |
| *4-cluster solution* | |  | |  | |  | |  | | |
| Kontos et al. 2012 (21) | | PCSS-22 | | Mean of 2.6 (range 1-7) days post injury | | - **CMF** (*headache, dizziness, fatigue, drowsiness, sensitivity to light sensitivity, to noise, feeling slowed down, mentally foggy, difficulty concentrating, difficulty remembering*) - **Affective** (s*adness, nervousness, feeling more emotional*) - **Somatic** (*nausea, numbness*) - **Sleep** (t*rouble sleeping, sleeping less than usual*) | | | | |
|  | |  | |  | |  | |  | | |
| *5-cluster solution* | |  | |  | |  | |  | | |
| Maruta et al. 2018-2 (28) | | Modified RPQ | | Mean of 5.8 (SD = 3.5) days post-injury | | - **Cognitive-fatigue** (*fatigue/tiring more easily, forgetfulness/poor memory, poor concentration, taking longer to think*) - **Vestibular** (*feeling of dizziness, balance problems*), oculomotor (*blurred vision, double vision*) - **Anxiety/mood** (*being irritable/easily angered, feeling depressed or tearful, feeling frustrated or impatient, restlessness*) - **Migraine** (*headaches, noise sensitivity/easily upset by loud noise, nausea and/vomiting, light sensitivity/easily upset by bright light*) | | | | |
|  | |  | |  | |  | |  | | |
| *7-cluster solution* | |  | |  | |  | |  | | |
| Heyer et al. 2017 (24) | | Symptom questionnaire | | Mean of 9.7 (SD = 7.8) days post-injury | | - **Dizziness-fogginess** *(feeling mentally foggy, difficulty remembering, balance problems, difficulty concentrating, lightheadedness)* - **Emotional** *(feeling sad, feeling more emotional, nervousness)* - **Cephalic** *(sensitivity to light, sensitivity to noise, nausea, irritability, headache)* - **Drowsiness** *(fatigue, drowsiness, sleeping more, feeling slowed down)* - **Somatic** *(numbness, neck pain, weakness)* - **Arousal-Stimulation** *(sleeping less, difficulty falling asleep)* - **Vomiting** *(vomiting)* | | | | |
|  |  |  |  |  | |  | | | | |

Abbreviations: CMF, Cognitive-Migraine-Fatigue; PCSS, post-concussion symptom scale; RPQ, Rivermead Post-concussion Symptoms Questionnaire; SD, standard deviation; SAC, Standardized Assessment of Concussion; SCAT3, Sport Concussion Assessment Tool 3.
