# Supplementary material for: Subtypes of Sport-Related Concussion: a Systematic Review and Meta-cluster Analysis
Source: Sports Med. 2020 Jul 27;50(10):1829–42. doi: 10.1007/s40279-020-01321-9 (PMC7497426; doi:10.1007/s40279-020-01321-9)
Supplement: Supplementary file 5 — Supplementary file5 (DOCX 42 kb) [file 40279_2020_1321_MOESM5_ESM.docx]

Subtypes of Sport-Related Concussion: a Systematic Review and Meta-Cluster Analysis

Sports Medicine

S Langdon MSc*^#^, M Königs PhD*, E A M C Adang BSc*, E Goedhart MD⁺, J Oosterlaan, PhD*

**Emma Children’s Hospital, Amsterdam University Medical Centers (location Academic Medical Center), Meibergdreef 9, 1105 AZ Amsterdam, The Netherlands.*

*^#^Corresponding author, e-mail address: s.langdon@amsterdamumc.nl*

⁺*Sport Medical Centre,* *Royal Dutch Football Association (KNVB), Woudenbergseweg 56-58, 3707 HX Zeist, The Netherlands.*

# **Online Resource 5 - SRC symptom clusters from literature matched to MCA-identified symptoms clusters**

| MCA identified SRC clusters |  | **Migraine cluster** | **Cognitive-emotional cluster** | **Sleep-emotional cluster** | **Neurological cluster** | **Undefined feelings cluster** |
| --- | --- | --- | --- | --- | --- | --- |
| Clusters from literature | |  |  |  |  |  |
| Heyer et al. 2017 (24) | **Emotional*** | 0% | 100% | 100% | 0% | 0% |
|  | **Dizziness-fogginess*** | 0% | 60% | 0% | 0% | 0% |
|  | **Cephalic*** | 80% | 25% | 25% | 0% | 0% |
|  |  |  |  |  |  |  |
| Howell et al. 2016 (29) | **Somatic** *(headache, pressure in head, neck pain, nausea or vomiting, sensitivity to light, sensitivity to noise)* | 57% | 0% | 0% | 43% | 0% |
|  |  |  |  |  |  |  |
| Howell et al. 2018 (30) | **Somatic** *(as described by Howell et al. 2016)* | 57% | 0% | 0% | 43% | 0% |
|  | **Cognitive** *(do not feel right, confusion, fogginess, difficulty concentrating, difficulty remembering)* | 0% | 60% | 0% | 0% | 40% |
|  | **Vestibular-ocular** *(vision problems, hearing problems/ringing, balance problems, dizziness)* | 0% | 0% | 0% | 25% | 0% |
|  |  |  |  |  |  |  |
| Lau et al. 2009 (31) | **Migraine** *(headache, nausea, vomiting, balance problems, dizziness, sensitivity to light/noise, numbness or tingling, visual problems)* | 43% | 0% | 0% | 29% | 0% |
|  | **Sleep** *(troubling sleeping, sleeping less than usual)* | 0% | 0% | 100% | 0% | 0% |
|  | **Cognitive** *(fatigue, drowsiness, feeling slowed down, fogginess, difficulty concentrating, difficulty remembering, sleeping more than usual)* | 0% | 57% | 14.3% | 0% | 0% |
|  |  |  |  |  |  |  |
| Lau et al. 2011 (45) | **Migraine** *(as described by Lau et al. 2009)* | 43% | 0% | 0% | 28.6% | 0% |
|  |  |  |  |  |  |  |
| Lau et al. 2012 (32) | **Migraine** *(as described by Lau et al. 2009)* | 43% | 0% | 0% | 28.6% | 0% |
|  | **Cognitive** *(as described by Lau et al. 2009)* | 0% | 52% | 14.3% | 0% | 0% |
|  |  |  |  |  |  |  |
| Sufrinko et al. 2017 (33) | **CMF** *(headache, dizziness, fatigue, drowsiness, sensitivity to light, sensitivity to noise ,feeling slowed down, fogginess, difficulty concentrating, difficulty remembering)* | 30% | 40% | 0% | 0% | 0% |
|  | **Sleep** *(trouble falling asleep, sleeping less than usual)* | 0% | 0% | 100% | 0% | 0% |
|  |  |  |  |  |  |  |
| Kontos et al. 2013 (39) | **PTM** *(headache, nausea, sensitivity to light, sensitivity to noise)* | 100% | 0% | 0% | 0% | 0% |
|  | **Sleep** *(trouble sleeping, sleeping more or less than usual)* | 0% | 0% | 50% | 0% | 0% |
|  |  |  |  |  |  |  |
| Mihalik et al. 2013 (43) | **PTM** *(as described by Kontos et al. 2013)* | 100% | 0% | 0% | 0% | 0% |
|  | **Headache** *(headache)* | 100% | 0% | 0% | 0% | 0% |
|  |  |  |  |  |  |  |
| Guty et al. 2018 (34) | **Headache** *(headache)* | 100% | 0% | 0% | 0% | 0% |
|  | **Sleep** *(fatigue, trouble falling asleep, sleeping less than usual, drowsiness)* | 0% | 0% | 100% | 0% | 0% |
|  |  |  |  |  |  |  |
| Teel et al. 2017 (46) | **Somatic early onset** (dizziness, headache, nausea, numbness/tingling, visual disturbances, vomiting) | 33% | 0% | 0% | 33% | 0% |
|  | **Cognitive** (fogginess, difficulty concentrating, difficulty remembering) | 0% | 100% | 0% | 0% | 0% |
|  | **Somatic evolving onset** *(drowsiness, fatigue, sensitivity to light, sensitivity to noise)* | 50% | 0% | 0% | 0% | 0% |
|  | **Neurobehavioral** *(difficulty sleeping, irritability, sadness, sleeping more than usual*) | 0% | 0% | 100% | 0% | 0% |
|  |  |  |  |  |  |  |
| Brett et al. 2018 (36) | **CMF** *(as described by Surfrinko et al. 2017)* | 30% | 40% | 0% | 0% | 0% |
|  |  |  |  |  |  |  |
| Cohen et al. 2018 (37) | **Somatic** *(vomiting, numbness)* | 0% | 0% | 0% | 50% | 0% |
|  | **Cognitive-fatigue-migraine** (*headache, dizziness, fatigue, drowsiness, sensitivity to light/noise, feeling slowed, down, fogginess, difficulty concentrating/remembering*) | 25% | 0% | 38% | 0% | 0% |
|  | **Affective** (*sadness, nervousness, feeling more emotional*) | 0% | 100% | 100% | 0% | 0% |
|  |  |  |  |  |  |  |
| Maruta et al. 2018-1 (38) | **Memory-attention** (e.g. *difficulty concentrating, forgetting names of people, difficulties with reading, writing and math*) | 0% | 25% | 0% | 0% | 0% |
|  | **Physical symptoms** (e.g. *having trouble staying awake, feeling cold, headache*) | 33% | 0% | 0% | 0% | 0% |
|  |  |  |  |  |  |  |
| Sufrinko et al. 2018 (44) | **PTM** *(as described by Kontos et al. 2013)* | 100% | 0% | 0% | 0% | 0% |
|  |  |  |  |  |  |  |
| Murdaugh et al. 2018 (40) | **Sleep** *(as described by Kontos et al. 2103)* | 0% | 0% | 100% | 0% | 0% |
|  | **Migraine** *(headache, nausea, vomiting, balance problems, dizziness, sensitivity to light, sensitivity to noise, numbness or tingling, visual problems)* | 44% | 0% | 0% | 22.2% | 0% |
|  | **Neuropsychological** (*irritability, sadness, nervousness and feeling more emotional*) | 0% | 75% | 100% | 0% | 0% |
|  |  |  |  |  |  |  |
| Churchill et al. 2017 (26) | **Cognitive*** | 0% | 66.7% | 0% | 0% | 33.3% |
|  | **Somatic*** | 55.6% | 0% | 0% | 44.4% | 0% |
|  |  |  |  |  |  |  |
| Kontos et al. 2016 (47) | **PTM** *(as described by Kontos et al. 2013)* | 100% | 0% | 0% | 0% | 0% |
|  |  |  |  |  |  |  |
| Paniccia et al. 2018 (41) | **Physical** (*headache, dizziness, balance problems, sensitivity to light/noise, clumsy, nausea, moving slowly and blurred vision*) | 43% | 0% | 0% | 14.3% | 0% |
|  | **Cognitive** (*difficulty concentrating/remembering, confused, fogginess, answers slowly and thinking slowly*) | 0% | 40% | 0% | 0% | 0% |
|  | **Fatigue** (e.g. *fatigue, sleeping more and drowsiness*) | 0% | 0% | 33% | 0% | 0% |
|  | **Emotional** (*irritability, feeling more emotional, sad and nervous*) | 0% | 100% | 100% | 0% | 0% |
| *See Online Resource 4 | | | | | | |
